# Trans-anal irrigation therapy to treat adult chronic functional constipation: systematic review and meta-analysis

**DOI:** 10.1186/s12876-015-0354-7

**Published:** 2015-10-16

**Authors:** Christopher D. Emmett, Helen J. Close, Yan Yiannakou, James M. Mason

**Affiliations:** Old Trust Headquarters, University Hospital of North Durham, North Road, Durham, DH1 5TW UK; School of Medicine, Pharmacy & Health, Durham University Queen’s Campus, University Boulevard, Thornaby, Stockton-on-Tees TS17 6BH UK; County Durham and Darlington NHS Foundation Trust, North Road, Durham, DH1 5TW UK

## Abstract

**Background:**

Trans-anal irrigation (TAI) is used widely to treat bowel dysfunction, although evidence for its use in adult chronic functional constipation remains unclear. Long-term outcome data are lacking, and the effectiveness of therapy in this patient group is not definitively known.

**Methods:**

Evidence for effectiveness and safety was reviewed and the quality of studies was assessed. Primary research articles of patients with chronic functional constipation, treated with TAI as outpatients and published in English in indexed journals were eligible. Searching included major bibliographical databases and search terms: bowel dysfunction, defecation, constipation and irrigation. Fixed- and random-effect meta-analyses were performed.

**Results:**

Seven eligible uncontrolled studies, including 254 patients, of retrospective or prospective design were identified. The definition of treatment response varied and was investigator-determined. The fixed-effect pooled response rate (the proportion of patients with a positive outcome based on investigator-reported response for each study) was 50.4 % (95 % CI: 44.3–56.5 %) but featured substantial heterogeneity (I^2^ = 67.1 %). A random-effects estimate was similar: 50.9 % (95 % CI: 39.4–62.3 %). Adverse events were inconsistently reported but were commonplace and minor.

**Conclusions:**

The reported success rate of irrigation for functional constipation is about 50 %, comparable to or better than the response seen in trials of pharmacological therapies. TAI is a safe treatment benefitting some patients with functional constipation, which is a chronic refractory condition. However findings for TAI vary, possibly due to varying methodology and context. Well-designed prospective trials are required to improve the current weak evidence base.

## Background

### Overview of the condition

Chronic constipation may be defined as ‘a symptom-based disorder defined as unsatisfactory defecation characterised by infrequent stools, difficult stool passage, or both, for at least three months’ [1]. For the purposes of this review, ‘chronic functional constipation’ refers to any condition fitting broadly within this definition, with no clear underlying cause. This includes obstructed defecation syndrome (ODS), functional defecation disorder (FDD), chronic idiopathic constipation (CIC), and constipation-predominant irritable bowel syndrome (IBS-C). This reflects the considerable overlap in symptoms between each of these conditions [2], and also the fact that observational studies indicate many patients reporting constipation do not fulfil the Rome III criteria for chronic functional constipation [1]. This definition does not include constipation secondary to a neurological cause (for example, spinal cord injury, stroke, Parkinson’s disease, Multiple Sclerosis), opioid-induced constipation or constipation secondary to any other medical diagnosis.

Chronic constipation is a common condition in the community: a recent systematic review [3] gave a pooled prevalence of 14 %, although it becomes more common in older people and women. There is a considerable burden of symptoms and decreased quality of life [1]: one recent study reporting ‘extremely/very bothersome’ symptoms in 72 % of IBS-C patients, 62 % of CIC patients with abdominal symptoms and 40 % of CIC patients without abdominal symptoms [2]. The costs of treating constipation are significant and appear to be increasing; one American study reported aggregate national (U.S.) costs of Emergency Department attendances due to constipation of $1.6 billion in 2011 [4].

### Trans-anal irrigation

Trans-anal irrigation therapy (TAI) is in widespread use throughout the UK as a treatment for bowel dysfunction. Irrigation involves instilling tap water into the rectum via the anus, using either a balloon catheter or cone delivery system. This is attached via a plastic tube to an irrigation bag holding up to 2 l of water; alternatively a low-volume system consisting of a hand pump and a cone may be employed. Low-volume systems deliver approximately 70 ml per irrigation; high-volume systems deliver up to 2 l of irrigation, although typically only 0.5–1.5 l is required. Patients vary in the frequency and volume of irrigation depending on their response to treatment; typically, irrigation is used 2–3 times per week. The low-volume system is cheaper, costing approximately £750 p.a. based on alternate-day use, compared with approximately £1400–1900 for high-volume irrigation, and may be more acceptable to patients. It is not known which system is more effective.

Irrigation has been used successfully to treat adults and children with neurogenic constipation [5–7], and faecal incontinence [8]. Proposed mechanisms of action include simple mechanical washout, colonic movement stimulated by the washout, or a combination of these [8]. However, evidence for the use of trans-anal irrigation therapy for chronic functional constipation in adults is not universally acknowledged, and there are questions about long-term benefit [9].

A review of current evidence for irrigation was undertaken to define what is known about this treatment as well as to identify areas where evidence is lacking and further research is required.

### Research question

What is the strength of the evidence for trans-anal irrigation therapy for chronic functional constipation, with reference to effectiveness, safety and methodological quality of studies?

## Methods

### Eligibility criteria

Primary research articles that include patients with chronic functional constipation as defined above, treated with retrograde trans-anal irrigation at home as outpatients, and published in English in indexed journals were eligible. The following were not eligible for inclusion: articles solely studying patients with a known cause for their constipation (e.g., neurogenic constipation, opioid-induced constipation, other organic cause); conference abstracts, audits, letters and commentaries; articles studying antegrade irrigation (Table 1). Reviews were not included but relevant review articles [8, 10] were screened for further relevant studies, as were citations of retrieved studies. No protocol was registered, however the review was reported in accordance with the PRISMA statement (2009) [11].

**Table 1:** Inclusion and exclusion criteria

Inclusion	Exclusion
Primary research	Audit/letters/commentaries/opinion/review articles
Patients with Chronic Functional Constipation (Obstructive defaecation and/or slow transit/IBS-C)	Studies in children (<18 years) only
Full articles published in peer-reviewed journals	Studies in neurogenic constipation only
English Language	Studies where all patients have undergone colorectal surgery (resection or rectopexy, etc.)
Retrograde irrigation using standard equipment performed at home	Studies in stoma patients only
Primary outcome is patient symptom improvement/response to treatment	Studies in antegrade irrigation only

### Search strategy

The following databases were systematically searched through Ovid Online:

- “All EBM Reviews” (comprising: Cochrane Database of Systematic Reviews (2005 to March 2015), ACP Journal Club (1991 to March 2015), Database of Abstracts of Reviews of Effects (1st Quarter 2015), Cochrane Central Register of Controlled Trials (March 2015), Cochrane Methodology Register (3rd Quarter 2012), Health Technology Assessment (1st Quarter 2015), NHS Economic Evaluation Database (1st Quarter 2015));
- Embase (1974–2015 Week 15);
- Ovid MEDLINE(R) (1946–April Week 2 2015).

The following search terms were used (searched in ‘all fields’): “bowel dysfunction”; “defaecation.”; “defecation”; “constipation”; “irrigation”. The Boolean Operators “AND” and “OR” were used to combine these terms appropriately and refine the search (Table 2). The search was limited to English language articles and to studies in humans.

**Table 2:** Search of bibliographic databases

Number	Searches	Results
1	Constipation.af^a^	90438
2	Bowel dysfunction.af	2264
3	Defecation.af	25606
4	Defaecation.af	1921
5	Irrigation.af	55773
6	1 OR 2 OR 3 OR 4	110886
7	5 AND 6	517
8	Limit 7 to English language	452
9	Limit 8 to Humans	405
10	Remove Duplicates from 9	292

^a^ *af* all fields (includes Subject headings and all test fields)

Abstracts and citations were screened by one researcher (CDE) and potentially relevant articles were retrieved. Articles that fulfilled the inclusion criteria were included in the review. Reference lists of eligible articles were searched to identify potentially relevant articles missed by the original database search. Another researcher (HJC) reviewed 10 % of the citations and abstracts, as well as 100 % of the full-text articles, to confirm appropriate implementation of the eligibility criteria and accuracy of data extraction. For practical and resource reasons a grey literature search was not performed, as the likelihood of finding appropriate studies not identified in retrieved citations or reviews was considered very small.

### Data collection

Data were extracted from eligible studies using standardised data collection forms. Data items included study methodology, patient information (including demographic details and definition of ‘constipation’ used), primary outcome data (including follow up period), duration of use of treatment, and adverse events reported. The Cochrane assessment of bias for non-randomised studies tool (ACROBAT-NRSI) [12] was used to evaluate methodological quality and sources of bias for the included studies.

### Outcomes

The primary outcome was the proportion of patients with an investigator-reported positive outcome to trans-anal irrigation therapy.

Secondary outcomes include response by constipation type, duration of treatment use and safety of treatment assessed by adverse event reporting in studies.

### Analysis

Both qualitative review of study results and quantitative analysis was performed. Rates of complications are reported and statistical pooling of proportion estimates was explored using fixed and random effect models within StatsDirect © Version 3. Both Q and I^2^ statistics were calculated to assess study heterogeneity. An Egger test was performed to assess risk of publication bias.

## Results

**Fig. 1:**
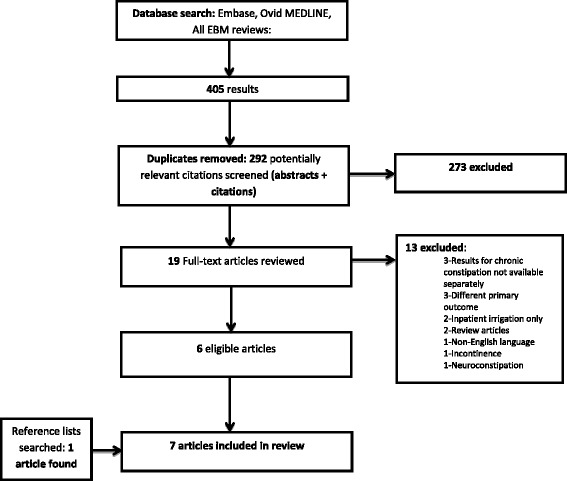
PRISMA flowchart. Flowchart showing number of abstracts and articles reviewed, numbers excluded, reasons for exclusion, numbers included in final analysis

Of 292 abstracts and citations reviewed, 19 full-text articles were retrieved. Of these, six were suitable to be included in the review [9, 13–17]. Reference lists of these articles were reviewed and a further eligible article was identified [18], giving a total of 7 articles (Fig. 1). All eligible studies reported outcomes using high-volume irrigation only. One further study using low-volume irrigation was found, not reporting constipation-specific outcomes and was excluded from the final analysis [19]. Studies identified were prospective cohort studies, or retrospective, uncontrolled case series from European nations (Table 3). In each study the patient case mix included patients with faecal incontinence, soiling and following colorectal surgery. However the articles reported outcomes separately for each group, making it possible to evaluate outcomes for chronic functional constipation. Reported mean duration of therapy varied from 8 months to 102 months (range 1–216 months across studies).

**Table 3:** Study characteristics

Study	Design and methods	Level of evidence^a^	Definition of constipation	Definition of successful treatment
Chan [13]	Prospective cohort study	III	Infrequent passage of stool +/− straining/ digitation/ incomplete emptying	i) → Improvement in Cleveland Constipation Score
				ii) → Patient-reported satisfaction
Christensen [9]	Retrospective questionnaire survey and case note review	III	Idiopathic constipation including slow transit, obstructed defecation and ‘undetermined’	i) → Ongoing use
				ii) → Resolved symptoms
				iii) → Still using irrigation at time of death
Koch [14]	Prospective cohort study	III	<2 bowel motions per week, straining or incomplete evacuation >50 % motions in previous year	Resolution of incomplete emptying or straining symptoms
Cazemier [15]	Retrospective case series questionnaire survey	III	Constipation according to Rome II criteria	Patient-reported satisfaction
Gosselink [16]	Retrospective case series, questionnaire survey	III	Obstructed defecation based on; straining, incomplete evacuation, digitation, fullness, <3 motions/ week	Patient-reported satisfaction
Gardiner [18]	Case series; not stated if prospective or retrospective	III	Obstructive defecation and slow transit (?which criteria used)	Patient-reported satisfaction
Crawshaw [17]	Retrospective case note review and questionnaire survey	III	The inability to evacuate the rectum when desired (includes obstructed defecation and dyssynergic defecation)	10 mm increase on VAS (10 % improvement)

^a^Eccles, Mason 2001 How to develop costconscious guidelines [25]

Studies were small, with an average number of patients per study of 36 (range 10–79); there was no evidence of a power calculation being performed for any study.

### Outcome of anal irrigation therapy

Patient-reported satisfaction, either subjective or using a visual-analogue scale, was the outcome most commonly reported (5 studies) [13, 15–18]. One study used resolution of symptoms as the outcome measure [14], another used a combination of patient-reported symptom improvement and ongoing use of treatment [9]. If a patient died while still using the treatment this was also considered successful. One study [13] reported both patient-reported satisfaction and change in Cleveland constipation score as markers of treatment success; the patient-reported satisfaction outcome was included in this analysis as it enabled meaningful comparison with other studies.

Studies report variable response rates to therapy (Table 4). The proportion of patients who had a positive outcome to therapy varied from 30 % [14] to 65 % [13, 16]. Overall, 254 patients with chronic functional constipation were included in studies, with 128 having a positive response to irrigation therapy (Table 4).

**Table 4:** Demographics and overall response to treatment

Study	Patients with Chronic Constipation (*n*)	Average age (Years)	Male:Female	Positive response *n*(%)	Time to assessment (Months (range))	Duration of therapy (Months (range))
Chan [13]	60	46	8:52	39 (65)	6^a^	10.7^a^
Christensen [9]	79	52^a^	25:62^a^	27 (34)	21 (1–116)^a^	8 (1–85)^a^
Koch [14]	10	55.4	4:7^a^	3 (30)	3^a^	-
Cazemier [15]	12	46	1:3	6 (50)	-	102 (30–216)^a^
Gosselink [16]	37^b^	54	5:32	24 (65)	56 (8–154)^a^	^d^
Gardiner [18]	41	-	-	21 (51)	-	-
Crawshaw [17]	15	54 (41–61)^a^	13:35^a^	8 (53)	12^ac^	-
Total	254	-	-	128		

^a^Whole cohort

^b^Obstructed Defaecation only

^c^Inferred from study report

^d^Not stated, but 73 % of patients still using TAI at 30 months

-Data not available

**Fig. 2:**
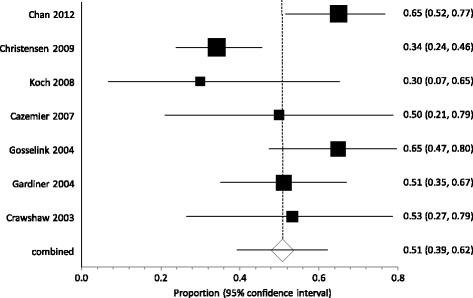
Proportion Meta-analysis plot [random effects] Forest plot showing response rates for each study, plus pooled response (diamond) with 95 % confidence intervals

A fixed effect analysis of proportions gave a pooled response rate of 50.4 % (95 % CI: 44.3–56.5 %). Although there was no evidence of publication bias (Egger: bias = 0.259, p = 0.91), there was evidence of substantially heterogeneity between studies (Q[6] = 18.2, p = 0.0057; I^2^ = 67.1 %). A random effects estimate was similar, if less precise: 50.9 % (95 % CI: 39.4–62.3 %), (see Fig. 2).

Four studies reported results for different sub-types of constipation (Table 5). Sample sizes in all studies were very small (10–37 patients with OD) and and differences between sub-groups remain anecdotal. When results from all four studies where results for different types of constipation are reported are combined, there was no consistent pattern of outcome between subtypes. Methodological weaknesses, inconsistencies in outcome measures and small sample sizes limit meaningful comparison.

**Table 5:** Risk of bias assessment

Risk of bias by type								
Study	Confounding	Selection	Measurement of interventions	Performance	Missing data	Measurement of outcomes	Reporting	Overall
Chan [13]	Moderate	Low	Low	Low	Moderate	Moderate	Low	Moderate
Christensen [9]	Moderate	Moderate	Moderate	No information	Low	Serious	Low	Serious
Koch [14]	Moderate	Low	Low	Low	Low	Moderate	Low	Moderate
Cazemier [15]	Serious	Serious	Serious	Low	Low	Serious	Low	Serious
Gosselink [16]	Low	Serious	Moderate	Low	Moderate	Serious	Low	Serious
Gardiner [18]	No information	No information	Moderate	No information	Low	Serious	No information	Serious
Crawshaw [17]	Moderate	Serious	Serious	Low	Serious	Serious	Low	Serious

### Safety of anal irrigation therapy

The most clinically significant risk associated with irrigation is bowel perforation. Only one study reported this complication [9] and this occurred in two patients. If reliably reported, this represents 2 perforations in approximately 110,000 irrigations, or less than 0.002 % risk per irrigation. No studies reported mortality associated with irrigation. Studies were inconsistent in their reporting of adverse events and the level of disaggregation between pathologies treated, thus only a narrative summary is possible.

Minor and self-limiting adverse events were commonplace in studies but may to some extent have been tolerated by patients, with up to 74 % of long term continuing users reporting some form of related and expected adverse events in one study [16]. The most commonly-reported adverse events included abdominal cramps/discomfort (33–40 %) [9, 15, 16]; anorectal pain (5–25 %) [9, 16]; anal canal bleeding (1–20 %) [9, 13]; leakage of irrigation fluid (30–75 %) [9, 16]; and expulsion of the rectal catheter (39 %) [9]. One study reports a 43 % incidence in ‘technical problems’ with irrigation [16]. In one study, 28 % of those discontinuing therapy gave side effects or technical issues with irrigation as a reason for discontinuing [9].

Therefore, whilst one or more side effects were experienced by a large proportion of patients undergoing anal irrigation, the risk of major life-threatening, life-limiting or irreversible complications was very low.

### Methodological quality

Generally, the studies were of weak methodology. There were no randomised controlled studies or case-controlled studies and most articles were retrospective questionnaire and case note based case series (Table 3). Two studies [13, 14] were prospectively designed with fixed follow up points, but numbers were relatively small (only 60 and 11 chronic functional constipation patients respectively). A further study [18] did not state whether data collection was prospective or retrospective.

Risk-of–bias assessment suggests that five studies were at serious risk of bias, and the other two were at moderate risk (Table 5). The retrospective questionnaire-based studies also suffered from non-response to surveys and missing data. This is likely to lead to bias and the results must be interpreted in light of this (i.e., were responders significantly more or less likely to have responded well to irrigation therapy?). Given the limitations of design and size, available studies are unable to provide robust evidence for the treatment effect of trans-anal irrigation.

Patient heterogeneity was also an issue. One study included both children and adult patients together [9] and the proportion of children was not reported. Neither was it stated whether there was a difference in outcome between the adults and children. One study [15] included three patients with neurological problems in its constipation cohort, representing 25 % of this study population. As neurogenic constipation may respond differently to irrigation [20], this may have affected the results. A further study included 5 patients out of 11 with chronic constipation who had had colorectal surgery (one resection and four rectopexies) [14]. Another study [17] also included patients who had undergone pelvic surgery or rectopexy in the chronic constipation cohort. It is not known precisely what effect these inclusions had on response to treatment but these remain a potential source of confounding.

## Discussion

This review brings together the findings of seven primary research studies which examine outcomes of trans-anal irrigation therapy in patients with chronic functional constipation.

Studies retrieved are small and not of robust methodological quality; only two are prospectively-designed, and there is the potential for reporting bias in the four studies that use questionnaires. This finding underlines the fact that the evidence for use of irrigation in functional constipation is currently weak.

The aggregate success rate of irrigation therapy is around 50 % based on these seven studies. Given the chronic and refractory nature of the symptoms in many of these patients this may be considered adequate, especially given the simple and reversible nature of the treatment [8]. By comparison, response rates for drug treatments in this group of patients has been reported as 20–40 %, though these are prospective RCTs reporting symptom based primary end-points [21–23]. Additionally, reported response rates in neurogenic constipation are only slightly higher-around 60 % [5]. Mean duration of use of treatment was reported between 8 months and 102 months. Inconsistencies in reporting findings, methodological differences and weak study design mean that there is insufficient evidence to state with any confidence exactly what the duration of benefit of treatment should be.

The majority of patients experience some form of adverse event although these are mostly minor, reversible and self-limiting. This may be a factor in determining the success of therapy: the need for high levels of patient motivation, as well as support from specialist nurses, is recognised [8]. The rates of life threatening complications are very low throughout the studies: Irrigation can be considered a safe therapy, when used with proper training.

There is insufficient evidence to state with any certainty how best to tailor therapy to patient symptoms. A recent review based on expert consensus [24] has proposed a number of regimes to overcome problems with irrigation and so improve outcomes, but experimental trial evidence is lacking, especially for functional constipation patients. In spinal cord injured patients, it has been found that emptying the rectosigmoid using irrigation stimulates colonic transit [24] however it is not clear whether this is transferable to patients with slow colonic transit and functional constipation. Scintigraphic studies have suggested that these patients have a different response to irrigation, with reduced colonic clearance compared with spinal cord injured patients [20]. In addition, none of the studies assess outcomes of low-volume anal irrigation systems.

Two previous systematic reviews examining trans-anal irrigation were found [8, 10]. These reviews, while valuable, have several limitations: They focus on irrigation as a therapy for several conditions including neurogenic constipation, faecal incontinence, idiopathic constipation and mixed symptoms; also, one review [10] incorporates studies of inpatient pulsed irrigation which is a very different therapy from home irrigation described in this review. The findings of this review are similar to the previous studies with respect to the weak nature of current evidence and the heterogeneity of the studies included. Subsequent to these reviews further studies have been identified and this review is the first to address irrigation therapy in idiopathic constipation only. This is also the first systematic review on this topic to be conducted in accordance with the PRISMA statement. Additionally, this is the first meta-analysis of the effectiveness of irrigation in chronic functional constipation.

## Conclusion

This review suggests that trans-anal irrigation may be an effective therapy for chronic constipation, and may be considered in patients who have not responded to medical management. Irrigation is safe and its effectiveness is at least comparable with pharmacological therapies. However, the evidence to guide its use in chronic functional constipation is weak, and its long-term benefits are unclear. There are no reported data on cost-effectiveness of irrigation: whether treatment provides good value for money from scarce health service resources. There is a clear need for well-designed prospective trials to evaluate the effectiveness, duration, and adverse consequences of treatment, as well as to assess how best to tailor therapy to individual patients. Future studies should have defined outcome measures, for example improvement in validated quality-of-life questionnaires within a defined time point. More evidence about the comparative effectiveness and cost-effectiveness of low-volume and high-volume irrigation systems would also be valuable.

## Abbreviations

TAI: Trans-anal irrigation
ODS: Obstructed defecation syndrome
FDD: Functional defecation disorder
CIC: Chronic idiopathic constipation
IBS-C: Irritable bowel syndrome (constipation-predominant)
PRISMA: Preferred reporting items for systematic review and meta-analysis
EBM: Evidence-based medicine
RCT: Randomised controlled trial

## References

[1] American College of Gastroenterology Chronic Constipation Task Force (2005). An evidence-based approach to the management of chronic constipation in north america. Am J Gastroenterol.

[2] Heidelbaugh JJ, Stelwagon M, Miller SA, Shea EP, Chey WD (2015). The spectrum of constipation-predominant irritable bowel syndrome and chronic idiopathic constipation: US survey assessing symptoms, care seeking, and disease burden. Am J Gastroenterol.

[3] Suares NC, Ford AC (2011). Prevalence of, and risk factors for, chronic idiopathic constipation in the community: systematic review and meta-analysis. Am J Gastroenterol.

[4] Sommers T, Corban C, Sengupta N, Jones M, Cheng V, Bollom A (2015). Emergency department burden of constipation in the United States from 2006 to 2011. Am J Gastroenterol.

[5] Ausili E, Focarelli B, Tabacco F, Murolo D, Sigismondi M (2010). Gasbarrini a, et al. Transanal irrigation in myelomeningocele children: an alternative, safe and valid approach for neurogenic constipation. Spinal Cord Off J Int Med Soc Paraplegia.

[6] Emmanuel A (2010). Review of the efficacy and safety of transanal irrigation for neurogenic bowel dysfunction. Spinal Cord Off J Int Med Soc Paraplegia.

[7] Faaborg PM, Christensen P, Kvitsau B, Buntzen S, Laurberg S, Krogh K (2009). Long-term outcome and safety of transanal colonic irrigation for neurogenic bowel dysfunction. Spinal Cord Off J Int Med Soc Paraplegia.

[8] Christensen P, Krogh K (2010). Transanal irrigation for disordered defecation: a systematic review. Scand J Gastroenterol.

[9] Christensen P, Krogh K, Buntzen S, Payandeh F (2009). Laurberg Sl. Long-term outcome and safety of transanal irrigation for constipation and fecal incontinence. Dis Colon Rectum.

[10] Tod AM, Stringer E, Levery C, Dean J (2007). Rectal irrigation in the management of functional Bowel Disorders : a Review. Br J Nurs.

[11] Moher D, Liberati A, Tetzlaff J, Altman DG (2014). Preferred reporting items for systematic reviews and meta-analyses. Ann Intern Med.

[12] Sterne J, Higgins JPT, Reeves BC (2014). A Cochrane risk of bias assessment tool : for Non- randomized studies of interventions (ACROBAT-NRSI).

[13] Chan DSY, Saklani A, Shah PR, Lewis M, Haray PN (2012). Rectal irrigation: A useful tool in the armamentarium for functional bowel disorders. Color Dis.

[14] Koch SMP, Melenhorst J, Van Gemert WG, Baeten CGMI (2008). Prospective study of colonic irrigation for the treatment of defaecation disorders. Br J Surg.

[15] Cazemier M, Felt-Bersma RJF, Mulder CJJ (2007). Anal plugs and retrograde colonic irrigation are helpful in fecal incontinence or constipation. World J Gastroenterol.

[16] Gosselink MP, Darby M, Zimmerman DDE, Smits AAA, van Kessel I, Hop WC (2005). Long-term follow-up of retrograde colonic irrigation for defaecation disturbances. Color Dis.

[17] Crawshaw AP, Pigott L, Potter MA, Bartolo DCC (2004). A retrospective evaluation of rectal irrigation in the treatment of disorders of faecal continence. Color Dis.

[18] Gardiner A, Marshall J, Duthie G (2004). Rectal irrigation for relief of functional bowel disorders. NursStand.

[19] Collins B, Norton C (2013). Managing passive incontinence and incomplete evacuation. Br J Nurs.

[20] Christensen P, Olsen N, Krogh K, Bacher T, Laurberg S (2003). Scintigraphic assessment of retrograde colonic washout in fecal incontinence and constipation. Dis Colon Rectum.

[21] Emmanuel A, Cools M, Vandeplassche L, Kerstens R (2014). Prucalopride improves bowel function and colonic transit time in patients with chronic constipation: an integrated analysis. Am J Gastroenterol.

[22] Johanson JF, Ueno R (2007). Lubiprostone, a locally acting chloride channel activator, in adult patients with chronic constipation: A double-blind, placebo-controlled, dose-ranging study to evaluate efficacy and safety. Aliment Pharmacol Ther.

[23] Quigley EMM, Vandeplassche L, Kerstens R, Ausma J (2009). Clinical trial: the efficacy, impact on quality of life, and safety and tolerability of Prucalopride in severe chronic constipation-a 12-week, randomized, double-blind, placebo-controlled study. Aliment Pharmacol Ther.

[24] Emmanuel AV, Krogh K, Bazzocchi G, Leroi AM, Bremers A, Leder D (2013). Consensus review of best practice of transanal irrigation in adults. Spinal Cord.

[25] Eccles M, Mason J (2001). How to develop cost-conscious guidelines.

